# Reliability, Validity and Acceptability of the PEDI-CAT with ASD Scales for Australian Children and Youth on the Autism Spectrum

**DOI:** 10.1007/s10803-024-06366-7

**Published:** 2024-04-28

**Authors:** Angela Chamberlain, Emily D’Arcy, Andrew JO Whitehouse, Kerry Wallace, Maya Hayden-Evans, Sonya Girdler, Benjamin Milbourn, Sven Bölte, Kiah Evans

**Affiliations:** 1 Curtin University, Perth, Australia; 2 Telethon Kids Institute, Perth, Australia; 3 Autism CRC, Brisbane, Australia; 4 Karolinska Institutet, Solna, Sweden; 5 The University of Western Australia, Perth, Australia; 6 Stockholm Health Care Services, Stockholm, Sweden

**Keywords:** Adolescents, Adaptive behavior, Assessment of functioning, Measurement, Overall utility, Psychometric properties

## Abstract

**Purpose:**

The PEDI-CAT (ASD) is used to assess functioning of children and youth on the autism spectrum; however, current psychometric evidence is limited. This study aimed to explore the reliability, validity and acceptability of the PEDI-CAT (ASD) using a large Australian sample.

**Methods:**

Caregivers of 134 children and youth on the spectrum participated in clinical assessments involving the administration of the PEDI-CAT (ASD), Vineland-3, PEDI-CAT (Original) and a feedback instrument. The PEDI-CAT (ASD) content was compared to the ICF Core Sets for ASD to summarize areas of functioning assessed and relevance to autism.

**Results:**

The PEDI-CAT (ASD) demonstrated good to excellent internal consistency and test-re-test reliability. Parallel forms reliability with the PEDI-CAT (Original) included significant correlations (good to excellent), however, *t*-tests showed significantly higher Social/Cognitive scores for the ASD version. Convergent validity results demonstrated that most PEDI-CAT (ASD) and Vineland-3 core domains were significantly correlated (poor to good). Content analysis revealed that the PEDI-CAT (ASD) covered less than half of the ICF Core Sets for ASD (mostly Activities and Participation codes). Just over half the codes assigned to the PEDI-CAT (ASD) were represented in the ICF Core Sets for ASD. Feedback on the acceptability of the measure was mixed, but overall was it was considered user-friendly and efficient.

**Conclusion:**

The PEDI-CAT (ASD) had adequate psychometric properties and acceptability as a measure of Activities and Participation codes. However, it lacks comprehensiveness and relevance when compared to the ICF Core Sets for ASD and has the potential to overestimate functioning.

**Supplementary Information:**

The online version contains supplementary material available at 10.1007/s10803-024-06366-7.

Autism spectrum disorder (ASD) is diagnosed based on specified challenges associated with social interaction and restricted, repetitive patterns of behaviour, interests, or activities impacting multiple areas of everyday life (American Psychiatric Association, 2013). Autism is characterised by a diverse collection of strengths and challenges leading to marked heterogeneity in functioning that is not only context-dependent but often fluctuating in nature (de Schipper et al., 2016; Mahdi et al., 2018a; Mahdi, Viljoen, Mahdi et al., 2018a, b). These diverse functional outcomes cannot be described by diagnosis alone and can create challenges for an equitable distribution of resources to meet individual support needs (Foster et al., 2016). Therefore, support planning and funding allocation should be informed by a comprehensive assessment of functioning (Commonwealth of Australia, 2013; National Institute for Health and Care Excellence, 2013; Whitehouse et al., 2018).

There is great interest in the development and validation of measures that assess functioning in individuals on the autism spectrum to ensure they are fit for purpose (Bölte et al., 2019; D’Arcy et al., 2022; Hayden-Evans et al., 2022; Kramer et al., 2012; McConachie et al., 2015; Whitehouse et al., 2018). However, little is known about the extent of use of these in practice. A recent study by D’Arcy et al. (2023) surveyed 98 clinicians in Australia and found that the most used measures were the Vineland Adaptive Behavior Scales (Sparrow et al., 2016; 53%) and the Adaptive Behavior Assessment System (Harrison & Oakland, 2015; 48%). The Pediatric Evaluation of Disability Inventory Computer Adaptive Test (PEDI-CAT; Haley et al., 2019) was reportedly used by 16% of clinicians (D’Arcy et al., 2023), and is a key measure in determining eligibility for funding in Australia (National Disability Insurance Agency, 2019). A modified version of the PEDI-CAT, termed the PEDI‐CAT (ASD), has recently been developed to address the specific needs of children and youth on the spectrum and their caregivers (Haley et al., 2019). However, its suitability to replace the original version as part of funding eligibility processes, particularly in the Australian context, has yet to be explored.

The PEDI-CAT (Original) and PEDI-CAT (ASD) both use computer adaptive test (CAT) methodology that applies an algorithm to select the most relevant items based on the individual’s responses to previous items, aiming to optimise test precision and efficiency (Haley et al., 2019). The ASD version takes into consideration the unique developmental trajectory associated with autism as well as the fluctuations in performance that can make it difficult to respond reliably to items (Coster et al., 2016; Haley et al., 2019; Kramer et al., 2012, 2015). This has been achieved through a series of qualitative and quantitative studies that have informed revisions to the item pool, instructions, and scoring (Haley et al., 2019).

Evidence for the structural validity of the PEDI-CAT (ASD) Daily Activities, Social/Cognitive and Responsibility domains has been demonstrated in a US study (Coster et al., 2016). Findings showed that criterion scores for children and youth on the spectrum were comparable to the PEDI-CAT (Original) criterion scores for children and youth without a disability, indicating they represent the same degree of functioning. Furthermore, confirmatory factor analysis and item fit analysis revealed that these PEDI-CAT (ASD) domains are unidimensional constructs. A subsequent study investigated test-retest reliability and concurrent validity of the PEDI-CAT (ASD) with the Vineland Adaptive Behavior Scales – Second Edition (Vineland-II; Sparrow et al., 2005) using a US sample of 39 parents of children and youth on the spectrum aged 10 to 18 years (Kramer et al., 2016). Intraclass correlation coefficient values showed excellent test-retest reliability for Daily Activities (0.92), Social/Cognitive (0.86) and Responsibility domain scores (0.90). Regarding concurrent validity, weak relationships were found between the PEDI-CAT (ASD) Daily Activities domain and the Vineland-II Communication (*r* = .25), Daily Living Skills (*r* = .57) and Socialization domains (*r* = .21). This suggests that the PEDI-CAT (ASD) can assess the practical aspects of daily life separate from the associated social and communication requirements, unlike the Vineland-II which assesses interpersonal skills within the Daily Living Skills items (Gleason & Coster, 2012). Stronger correlations were found between the PEDI-CAT (ASD) Responsibility domain and the Vineland-II Communication (*r* = .69), Daily Living Skills (*r* = .70) and Socialisation (*r* = .72) domains. These findings highlight the interpersonal skills required for many Responsibility items. The study also found stronger than expected correlations between the PEDI-CAT (ASD) Social/Cognitive domain and the Vineland-II Communication (*r* = .81) and Socialisation (*r* = .65) domains, considering that the PEDI-CAT (ASD) rates performance with communication supports while the Vineland-II does not. However, this difference may have had a limited impact in this study due to the verbal abilities of the sample. Overall, the study concluded that the PEDI-CAT (ASD) was an appropriate alternative to the Vineland-II and may be more suitable for assessing specific functional domains, especially the management of tasks essential for independent living (Kramer et al., 2016).

Preliminary evaluation of the content validity of the PEDI-CAT (ASD) has recently been conducted (D’Arcy et al., 2022) by comparing items to the World Health Organization’s International Classification of Functioning, Disability and Health (ICF) using an established linking methodology (Cieza et al., 2019). Analysis revealed that 94% of the codes assigned to the PEDI-CAT (ASD) were distributed in the Activities and Participation domain of the ICF. The remaining assigned codes were represented in the Body Functions domain; the measure was not found to assess the Environmental Factors domain. Coverage across the Activities and Participation domain was inconsistent, with nearly three quarters of assigned codes distributed across only two of the nine chapters within the domain (36% in the Mobility and 26% in the Self-care chapters). The remaining chapters had a representation of 1–10%. Further investigation of the distribution of assigned codes *within* the chapters would provide additional information regarding the comprehensiveness of the PEDI-CAT (ASD) and reveal gaps in coverage.

To determine the relevance of the PEDI-CAT (ASD) for children on the spectrum, the study by D’Arcy et al. (2022) linked the measure to the ICF Core Set for Autism (Autism ICF-CS) for 0 to 5-year-olds (Autism 0–5 ICF-CS; Bölte et al., 2014; Bölte et al., 2019). Results showed that 65% of the codes assigned to the PEDI-CAT (ASD) were not represented in the Autism 0–5 ICF-CS (D’Arcy et al., 2022), suggesting a large proportion of it is not relevant to young children on the spectrum.

However, the findings should be interpreted with caution, as the analyses were conducted using the full item bank when, in practice, only a subset of PEDI-CAT (ASD) items are administered due to the CAT technology (Haley et al., 2019). This may have resulted in an overestimation of comprehensiveness and underestimation of relevance, and further analysis based on the items administered for a representative sample has been recommended (D’Arcy et al., 2022). As the PEDI-CAT (ASD) is designed to assess individuals from 0 to 21 years of age, comparison against the Autism ICF-CS for each age group (0–5, 6–16 and 17 + years) using sample data would provide more accurate and complete depiction of the validity of its content.

Whilst there are numerous established measures of functioning, a recent review of 13 measures suitable for assessing children and youth on the spectrum concluded that most had only adequate overall clinical utility and were insufficient if used as the only method of collecting information about functioning, including the PEDI-CAT (ASD) (Hayden-Evans et al., 2022). Of note, however, researchers providing the psychometric evidence were also often involved in the development of the measures (Hayden Evan et al., 2022). Thus, this study sought to provide an independent evaluation of a range of measurement properties from a broad sample across several age groups, in an Australian context. Specifically, it aimed to explore: (1) Reliability by evaluating internal consistency, test-retest reliability, and parallel forms reliability to allow comparison with the PEDI-CAT (Original) to determine how similar / different they are; (2) Validity through comparison with the most recent edition of the Vineland Adaptive Behavior Scale (Vineland-3; convergent validity) and the Autism ICF-CS (content validity); and (3) Acceptability from the perspective of caregivers (as the informants).

## Methods

This mixed methods study was conducted in accordance with the Australian Code for the Responsible Conduct of Research (National Health and Medical Research Council 2018a) and the National Statement on Ethical Conduct in Human Research 2007 - Updated 2018 (National Health and Medical Research Council 2018b). Ethical approval was received by Bellberry Human Research Ethics Committee (2018-10-852).

### Procedures

#### Recruitment

This study was part of a larger project designed to investigate psychometric properties across a range of measures (Evans et al., 2022 - Supplementary File contains the full data collection protocol). Caregivers of individuals under 21 years of age with an autism diagnosis, who were enrolled in Australia’s National Disability Insurance Scheme (NDIS), were invited to participate. Recruitment Wave 1 involved the agency that administers the NDIS forwarding an invitation letter via post or email to caregivers living in four Australian states: Queensland, New South Wales, Victoria or Western Australia. Recruitment Wave 2 was undertaken as the study progressed, to boost sample sizes for measuring parallel forms reliability and target recruitment of older adolescents / young adults. In this situation, families in Western Australia (where the research team was based) were recruited via professional networks, research databases and advertisements on websites/social media.

**Fig. 1:**
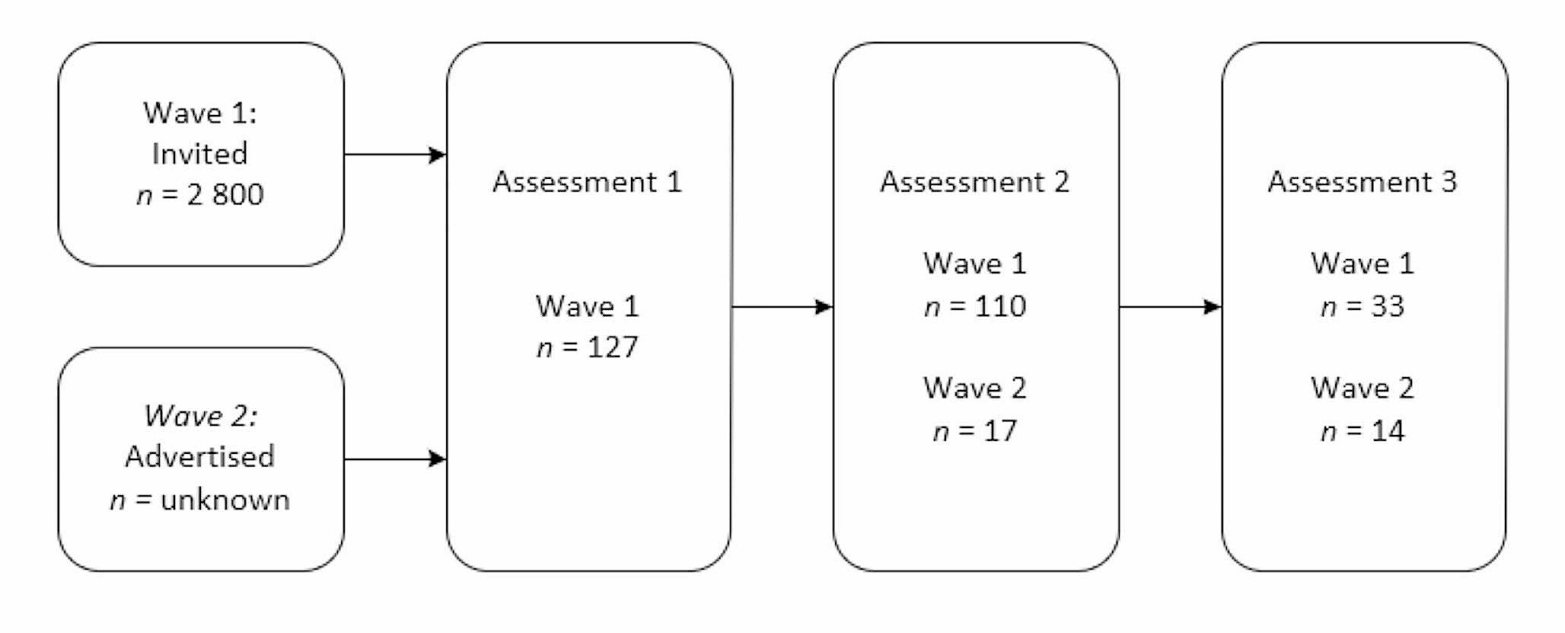
Flow chart of participant sample size (n) for each recruitment wave and project phase

All participants recruited via Wave 1 were scheduled to complete Assessment 1 and 2, while those recruited via Wave 2 were streamed directly into Assessment 2 and 3 (Fig. 1). Participants were stratified based on the child’s or youth’s age (0–5 years, 6–10 years, 11–16 years, 17–20 years) and geographical location (state).

#### Clinical Assessments

Assessment 1 involved administration of the PEDI-CAT (ASD) and Vineland-3 *Parent/Caregiver* Form to enable internal consistency and convergent validity to be determined. Assessments were conducted by a professional with tertiary qualifications in a health and/or disability field. Where possible, the assessment occurred within a clinical setting, substituted by a teleconference meeting, home visit or remote administration (Vineland-3) if required.

Assessment 2 was completed between one and five weeks after Assessment 1. It involved the repeat administration of the PEDI-CAT (ASD) to collect data for test-retest reliability. This was either completed with the same clinic and assessor or in the home environment with an occupational therapist (OT) as part of a comprehensive assessment that included the Vineland-3 *Interview* Form for convergent validity. The Vineland-3 Interview Form was completed over the telephone (audio recorded) within 14 days of the home visit.

Assessment 3 involved the administration of the PEDI-CAT (Original) to establish parallel forms reliability, and/or a semi-structured interview to explore the acceptability of the PEDI-CAT (ASD). It was conducted by an OT via videoconference (recorded) approximately 3 weeks after Assessment 2. Caregivers were also given an opportunity to provide feedback via an online REDCap survey link without completing the PEDI-CAT (Original). The interview/survey questions are presented in Supplementary File S1 - Table 1.

**Table 1:** Internal consistency of the PEDI-CAT (ASD) (*n* = 113)

Domain					95% CI		
	No. items	*M*	*SD*	ω	*LL*	*UL*	*SE*
Daily Activities	8	2.96	1.06	0.92	0.90	0.94	0.01
Mobility	16	3.01	1.20	0.89	0.83	0.93	0.02
Social/Cognitive	12	2.39	1.12	0.90	0.86	0.92	0.01
Responsibility	16	1.91	1.06	0.93	0.89	0.95	0.02

Note. ω = McDonald’s omega; CI = confidence interval; *LL =* lower limit; *UL* = upper limit; *SE* = standard error; ω = 0.80 − 0.89 = good; ω > 0.90 = excellent

### Participants

Caregivers of 134 children and youth with an autism diagnosis were enrolled in the study during 2019. A total of 117 were recruited via Wave 1 and completed Assessment 1, with 100 completing Assessment 2. Caregivers of 17 children and youth commenced at Assessment 2 as part of Wave 2. Finally, caregivers of 47 children and youth participated in Assessment 3, 25 of whom completed the PEDI-CAT (Original). Families who did not complete their scheduled assessments either withdrew, could not be contacted or were unable to complete the assessments within specified timeframes. Some assessment data were also identified as invalid and excluded during the data checking processes. Thus, data from the full sample were not available for every analysis.

In total, 130 caregivers provided information (this figure is less than the number of youth due to four families having two siblings participate in the study). Most were biological mothers (90%) and had a mean age of 42 years. The children and youth were predominantly male (72%) and ranged in age from 3 to 18 years (*M* = 9 years 6 months). At least one co-occurring condition was present in 60% of cases, the most common being attention deficit hyperactivity disorder (32%), intellectual disability (23%) and communication disorders (22%). Participant characteristics are detailed in Supplementary File S1 - Table 2. Of the 108 children and youth for whom autism diagnostic information was provided, almost half met DSM-5 level 2 criteria with 45% requiring substantial support for social communication and 49% requiring substantial support for restricted, repetitive behaviors (Supplementary File S1 - Table 3).

**Table 2:** Test-retest reliability of the PEDI-CAT (ASD) (*n* = 92)

Domain		95% CI		*F* value
	ICC	*LL*	*UL*	
Daily Activities	0.92 **	0.88	0.95	24.50
Mobility	0.92 **	0.88	0.95	23.56
Social/Cognitive	0.90 **	0.85	0.93	18.89
Responsibility	0.89 **	0.84	0.93	17.18

Note. ICC = intraclass correlation coefficient; CI = confidence interval; *LL* = lower limit; *UL* = upper limit; ICC = 0.75 − 0.90 = good; ICC > 0.90 = excellent.

**p < .01, two-tailed

**Table 3:** pearson’s r correlations, paired t-tests and limits of agreement between the PEDI-CAT (ASD) and PEDI-CAT (Original) (*n* = 23)

Domain	t-Tests					95% LOA	
	*r*	*t*	*M* (Autism)	*M* (Original)	*M* (Difference)	*LL*	*UL*
Daily Activities	0.94**	-0.24	30.24	30.46	-0.22	-8.57	8.14
Mobility	0.94**	-0.19	29.67	29.96	-0.28	-13.96	13.39
Social/Cognitive	0.91**	4.53**	32.57	28.11	4.46	-4.79	13.70
Responsibility	0.86**	-0.35	37.70	38.00	-0.30	-8.46	7.85

*Note. M* (Autism) = mean of PEDI-CAT (ASD); *M* (Original) = mean of PEDI-CAT (Original); LOA = Limits of Agreement; *LL* = lower limit; *UL* = upper limit; *r* = .75 − .90 = good; *r* > .90 = excellent

**p < .01, two-tailed

The socio-economic status of participants was estimated using the Australian Bureau of Statistics Socio-Economic Indexes for Areas (SEIFA) Index of Relative Socio-Economic Advantage and Disadvantage (IRSAD; Australian Bureau of Statistics, 2018b). To determine IRSAD, participant postcodes were assigned a decile rank. The median IRSAD decile was 8 (range = 1–10), suggesting that most participants lived in areas associated with high socio-economic advantage and low socio-economic disadvantage. Postcodes also allowed for categorization of remoteness (Australian Bureau of Statistics, 2018a). Most participants (91%) resided in Major Cities of Australia, with 6% living in Inner Regional Australia and 3% in Outer Regional Australia.

### Measures

#### PEDI-CAT (Original) and PEDI-CAT (ASD)

The PEDI-CAT (Original) (Haley et al., 2019) is a caregiver-report measure of functioning for young people under 21 years of age. Functioning is measured using a bank of 276 items across four domains: Daily Activities, Mobility, Social/Cognitive, and Responsibility. The Daily Activities, Mobility and Social/Cognitive domains assess performance in specified activities (in a typical environment with usual supports), rated according to level of difficulty (i.e., “unable”, “hard”, “a little hard”, “easy”, or “I don’t know”). The Responsibility domain measures independence by determining the extent to which the child or youth assumes responsibility in life tasks, rated on a 5-point scale ranging from “adult/caregiver has full responsibility; the child does not take any responsibility” to “child takes full responsibility without any direction, supervision or guidance from an adult/caregiver”. The items presented are customised based on responses and a software algorithm is used to determine the minimum number of items required to generate a score. For the Content-Balanced version (used in this study) approximately 120 items from the total bank (30 per domain) are administered during a single assessment (Haley et al., 2019). The Windows application of the measure was used for this study as the web-based version had not yet been released.

The PEDI-CAT (ASD) is a modified version for children and youth on the spectrum (Haley et al., 2019). Modifications include: eight new Daily Activities items; eight new and 11 revised Social/Cognitive items; eight new and/or revised Responsibility items; modified instructions including item-specific directions to further define the task, behaviour, context or supports permitted (including communication devices); adjustment to scoring (Social/Cognitive domain); and revised item maps (Haley et al., 2019). The manual states that the PEDI-CAT (ASD) takes approximately 12 to 17 min to complete; in this study, mean completion times ranged between 23 and 29 min (greatest for Assessment 1).

#### Vineland Adaptive Behaviour Scales-3

The Vineland-3 is a measure of adaptive behaviour for individuals with intellectual, developmental and other disabilities (birth to 90 + years; Sparrow et al., 2016). It is comprised of three core domains (Communication, Daily Living Skills, and Socialization), and two optional domains (Motor Skills and Maladaptive Behavior). For the current study, the Comprehensive version was administered and scored online using Q-Global (Pearson Clinical Assessment, 2022). It consists of a total item pool of 381 core items and 121 optional items, however, only a subset of items relevant to the individual’s developmental level is presented (excluding the Maladaptive Behaviors domain, for which all items are administered). These items are determined using start points and basal and ceiling rules. Item responses are scored according to the frequency of the behaviour (i.e., “usually/regularly” = 2, “sometimes” = 1, or “does not” present behaviour = 0). For this study, the Parent/Caregiver Form was completed independently by caregivers (mean completion time = 50–57 min, greatest for Assessment 1) and the Interview Form was administered and interpreted by an OT during an interview with the caregiver (mean time = 89 min).

### Data Analysis

All data were managed and stored through the secure web application, REDCap (Harris et al., 2009, 2019 ). Data were imported into R (The R Foundation, n.d.) and/or SPSS (IBM Corp, 2020) for statistical analyses, or NVivo (QSR International Pty Ltd., 2020) for qualitative analysis. An alpha of 0.05 was selected to determine statistical significance.

#### Internal Consistency

The internal consistency (homogeneity) of PEDI-CAT (ASD) items was calculated using McDonald’s omega (ω; McDonald, 1999). The omega was selected as the PEDI-CAT (ASD) was developed using item response theory and uses software algorithms to select items to be administered from hierarchically structured item pools (Haley et al., 2019), therefore did not meet the assumptions necessary for coefficient alpha (Dunn et al., 2014). The magnitude of the internal consistency coefficient is described as: < 0.70 = unacceptable, 0.70 – 0.79 = fair, 0.80 – 0.89 = good and > 0.90 = excellent (Cicchetti, 1994).

#### Test-retest Reliability

Test-retest reliability was determined by examining the consistency of PEDI-CAT (ASD) domain T*-*scores across repeated administrations. Assessment 1 scores were correlated with Assessment 2 scores using intraclass correlation coefficients (ICC) based on single-measures (type), absolute-agreement (definition) and a two-way mixed effects model (Koo & Li, 2016). The magnitude of the ICC is described according to the following categories, where the lower bound confidence interval (CI) is referenced as the beginning range of reliability: < 0.50 = poor, 0.50 to 0.75 = moderate, 0.75 to 0.90 = good and > 0.90 = excellent (Koo & Li, 2016).

#### Parallel Forms Reliability

To explore parallel forms reliability, PEDI-CAT (ASD) domain scores from Assessment 2 were compared with PEDI-CAT (Original) domain scores from Assessment 3 by calculating Pearson product-moment correlations (Pearson’s *r*; 2-tailed), paired-samples *t*-tests and limits of agreement (LOA). The magnitude of the correlation coefficient is described as: < 0.50 = poor, 0.50 to 0.75 = moderate and > 0.75 = good (Portney & Watkins, 2009).

#### Convergent Validity

Convergent validity was determined by measuring the strength of association between the PEDI-CAT (ASD) domain T-scores and the Vineland-3 domain standard scores for the Parent/Caregiver and Interview Forms (Communication, Daily Living Skills, Socialization and Motor Skills domains). Pearson’s *r* was calculated, and the correlation coefficient magnitude is described according to the following criteria: < 0.50 = poor, 0.50 to 0.75 = moderate and > 0.75 = good (Portney & Watkins, 2009).

#### Content Validity

To evaluate content validity, linking data from a related study were utilized (D’Arcy et al., 2022). This linking data was obtained by mapping the PEDI-CAT (ASD) full item banks against the ICF in accordance with established ICF linking methodology (Cieza et al., 2019), resulting in a list of ICF codes that captured the content of the measure. The list was then simplified by reducing three and four level codes to second level (parent) codes. If an item was assigned two codes that both fell under the same parent code (e.g., d5600 and d5601 both fall under d560) the duplicate code was removed. In the current study, this linking data was analyzed to compare the content of the PEDI-CAT (ASD) to the ASD ICF-CS. To provide a summary of the areas of functioning assessed by the PEDI-CAT (ASD) and identify gaps in coverage (comprehensiveness), frequency distributions and percentages were calculated by tallying the number of ASD ICF-CS codes assigned to the PEDI-CAT (ASD). To determine how much of the PEDI-CAT (ASD) assessed areas of functioning pertinent to autism according to the ASD ICF-CS (relevance), the percentage of assigned ICF codes that were represented in the ASD ICF-CS was calculated. Analysis was first conducted for the full item bank of both the original and ASD versions for comparison. A second analysis was conducted using the actual items administered from the PEDI-CAT (ASD) during Assessment 1, to reflect the CAT nature of the measure in clinical practice. The sample was divided into subsamples (0–5, 6–11, 12–16 and 17 + years) to allow comparison of the administered content to the ASD ICF-CS by age groups: 0–5 years (ASD 0–5 ICF-CS), 6–16 years (ASD 6–16 ICF-CS) and 17 + years (ASD 17 + ICF-CS). The PEDI-CAT (ASD) items and associated ICF codes administered to each participant were compared to the full and age-specific ASD ICF-CS to provide a percentage of the ASD ICF-CS assessed for each participant.

#### Acceptability

To explore the acceptability of the PEDI-CAT (ASD), caregiver responses were initially coded in NVivo, then thematically analyzed to identify common ideas present in the data (DePoy & Gitlin, 2015). Emerging themes were repeatedly reviewed and modified as new information was revealed, and findings were confirmed through discussion with two additional researchers.

## Results

### Reliability

#### Internal Consistency

McDonald’s omega was calculated for each domain from 113 assessments administered during Assessment 1. Results ranged from 0.89 to 0.93, indicating good to excellent internal consistency (Table 1).

#### Test-retest Reliability

A total of 92 cases from Assessment 1 were compared with 92 cases from Assessment 2 (*M* = 19 days between assessments). The ICC values ranged from 0.89 to 0.92 (*p* = < 0.01), with lower bound CIs commencing at 0.84, indicating good to excellent test-retest reliability (Table 2).

#### Parallel Forms Reliability

A total of 23 cases were analyzed to compare the PEDI-CAT (ASD) with the PEDI-CAT (Original), completed a mean of 23 days later. Correlation coefficients were all statistically significant and classified as good to excellent in strength (*r* = .86 to 0.94, *p* = < 0.01). Results from the *t*-tests demonstrated significantly higher scores for the PEDI-CAT (ASD) Social/Cognitive domain (*p* = < 0.01). The mean difference in scores for all other domains were non-significant. Limits of agreement were all within one standard deviation of the mean on the norm-referenced scale (where *M* = 50 and *SD* = 10), except the upper limit for the Social/Cognitive domain which was between one and two standard deviations above the mean. Correlations, *t*-tests and LOA are presented in Table 3. Bland-Altman plots (Bland & Altman, 1986) visually demonstrate a relationship between the two versions and the absence of a systematic bias in measurement error (Supplementary File S1 - Fig. 1).

### Validity

#### Convergent Validity

To examine the convergent validity of the PEDI-CAT (ASD) and the Vineland-3 Parent/Caregiver Form, a total of 110 cases from Assessment 1 were analyzed (Table 4). All correlation coefficients were statistically significant. The only relationship classified as good in strength was between the PEDI-CAT (ASD) Responsibility and Vineland-3 Daily Living Skills domains (*r* = .77, *p* = < 0.01). More than half (60%) of the statistically significant relationships were moderate in strength (*r* = .51 to 0.74, *p* = < 0.01) and the remainder (35%) were poorly correlated (*r* = − .25 to 0.48, *p* = < 0.05 to < 0.01).

**Table 4:** Pearson’s r correlations between PEDI-CAT (ASD) and vineland-3 parent/caregiver form

	Vineland-3 Parent/Caregiver Form domain				
PEDI-CAT (ASD) domain	Adaptive Behavior Composite	Communication^a^	Daily Living Skills^a^	Socialization^a^	Motor^b^
Daily Activities	0.55**	0.43**	0.58**	0.51**	0.43**
Mobility	0.48**	0.35**	0.48**	0.51**	0.43**
Social/Cognitive	0.72**	0.67**	0.65**	0.71**	0.62**
Responsibility	0.74**	0.63**	0.77**	0.67**	0.46**

*Note. r* < .50 = poor; *r* = .50 − .75 = moderate; *r* > .75 = good

^a^*n* = 110. ^b^*n* = 69

*p < .05, two-tailed. **p < .01, two-tailed

The PEDI-CAT (ASD) and the Vineland-3 Interview Form were compared using a total of 66 cases from Assessment 2 (Table 5). No statistically significant relationships were found between the PEDI-CAT (ASD) Responsibility domain and the Vineland-3 Motor domain. Of the statistically significant relationships, over half (53%) were moderate in strength (*r* = .50 to 0.70, *p* = < 0.01) and the remainder (47%) poorly correlated (*r* = .30 to 0.49, *p* = < 0.05 to < 0.01).

**Table 5:** Pearson’s r correlations between PEDI-CAT (ASD) and vineland-3 interview form

	Vineland-3 Parent/Caregiver Form domain				
PEDI-CAT (ASD) Domain	Adaptive Behavior Composite	Communication^a^	Daily Living Skills^a^	Socialization^a^	Motor^b^
Daily Activities	0.54**	0.49**	0.56**	0.30*	0.57**
Mobility	0.41**	0.30*	0.40**	0.30*	0.40*
Social/Cognitive	0.64**	0.60**	0.46**	0.51**	0.45**
Responsibility	0.70**	0.62**	0.61**	0.50**	0.33

*Note. r* < .50 = poor; *r* = .50 − .75 = moderate; *r* > .75 = good

^a^*n* = 66. ^b^*n* = 34

*p < .05, two-tailed. **p < .01, two-tailed

#### Content Validity

Frequency distributions were calculated using the full item banks of the PEDI-CAT (ASD) and PEDI-CAT (Original). Results revealed that the measures covered a respective 42% and 41% of the codes in the Autism ICF-CS, with the ASD version covering one more code (d331 pre-talking) than the original version. For the PEDI-CAT (ASD), 75% of Activities and Participation, 10% of Body Functions and Structure and no Environmental Factors codes were represented (Supplementary File S1 - Table 4).

Further analysis of the PEDI-CAT (ASD) Activities and Participation domain (Supplementary File S1 - Table 5) showed that Chap. 5 Self-care codes were covered with the greatest frequency (72 times), with each of the seven codes therein covered between five times (d530 Toileting) and 19 times (d540 Dressing). Chapter 3 Communication codes were covered 22 times, with each of the seven codes therein covered between one time (d310 Communicating with - receiving - spoken messages and d331 Pre-talking) and seven times (d330 Speaking) and Chap. 6 Domestic Life codes were covered 21 times, with every code covered at least once. The chapters with the least frequently covered codes were Chap. 9 Community, Social and Civic Life codes with two of the three codes covered once each (d910 Community life and d920 Recreation and leisure); Chap. 4 Mobility with both codes covered two times (d475 Driving) and four times (d470 Using transportation), and Chap. 8 Major Life Areas with three of eight codes covered between one time (d860 Basic economic transactions) and five times (d880 Engagement in play).

The PEDI-CAT (ASD) was assigned 350 ICF codes, 189 (54%) of which were represented in the ASD ICF-CS. This revealed an overall increase in relevance compared to the PEDI-CAT (Original), which was assigned 321 ICF codes, with 166 (52%) represented in the Autism ICF-CS. Comparison at the domain level showed that the PEDI-CAT (ASD) Social/Cognitive was 2% less relevant to the ASD ICF-CS than the PEDI-CAT (Original) (81% and 83%, respectively); and the Daily Activities and Responsibility domains retained a relevance of 71% and 98%, respectively, for both PEDI-CAT versions (Supplementary File S1 - Table 6).

The content from 113 PEDI-CAT (ASD) assessments administered during Assessment 1 were analyzed according to subsamples based on age. For participants aged 0–5 years (*n* = 26), a median of 23% (range 22–26%) of the Autism 0–5 ICF-CS was covered during the assessments. For the 6–11 (*n* = 57) and 12–16 years subsamples (*n* = 28), a median of 26% (22–30%) and 26% (23–28%) of the Autism 6–16 ICF-CS were assessed, respectively. The 17 + years subsample was not analyzed due to the small sample size (*n* = 2). When administered items were compared against the full Autism ICF-CS, median percentages were higher (ranging from 27 to 29% across the subsamples), demonstrating greater relevance than age-specific Autism ICF-CS.

The administered PEDI-CAT (ASD) items covered medians of 49–53% of the full ICF-CS Activities and Participation codes. For age specific ICF-CS, 55% of the Autism 0–5 ICF-CS, and 56% of the Autism 6–16 ICF-CS Activities and Participation codes were covered, with 5–6% of Body Functions and no Environmental Factors codes represented. The percentage of the Autism ICF-CS covered according to subsamples can be seen in Supplementary File S1 – Tables 7, 8 and 9.

### Acceptability

Feedback on the acceptability of the PEDI-CAT (ASD) was provided by 43 caregivers via interview (*n* = 20) and/or online survey (*n* = 23). Most caregivers reported that the PEDI-CAT (ASD) results were as expected. The information either confirmed or increased their understanding of the child’s or youth’s functioning and was useful in planning support needs. Other caregivers expressed concerns regarding the PEDI-CAT (ASD) accuracy in measuring all aspects of functioning relevant to autism, fluctuations in functioning across environments and the level and nature of support required for success. See Supplementary File S1 for further information on the themes of accuracy and usefulness.

Aspects of the PEDI-CAT (ASD) that caregivers liked and disliked are presented in Supplementary File S1 - Table 10. Caregivers commented favourably on this study’s assessment process, stating that the availability of an assessor for clarification when competing the PEDI-CAT (ASD) was helpful.

## Discussion

Reliability of the PEDI-CAT (ASD) was evaluated in relation to internal consistency, test-retest reliability and parallel forms reliability. Internal consistency was described as good to excellent, suggesting homogeneity of items. Test-retest reliability revealed good to excellent stability over the test-retest period, congruent with previous research (Kramer et al., 2016). While parallel forms reliability results showed that the PEDI-CAT (ASD) and PEDI-CAT (Original) domains had good to excellent correlations, results from the *t*-tests found that the Social/Cognitive domain scores were significantly higher on the PEDI-CAT (ASD), indicating that it specifies higher levels of social and cognitive functioning than the original version. This finding is not surprising given that the Social/Cognitive domain underwent the greatest revisions during the development of the ASD version, including adjustments to items, instructions, the software algorithm, and scaled scores (Haley et al., 2019). Revisions to the CAT parameters were made to this domain after research demonstrated consistent differences in the responses of caregivers of children and youth on the spectrum and the standardized population in more than half of the Social/Cognitive items (Coster et al., 2016), likely a reflection of the unique pattern of strengths and challenges associated with autism and/or the caregivers’ interpretation of these (Kramer et al., 2015). The revisions ensured criterion scores were a valid representation of functioning while remaining comparable to other groups assessed using the original version (Coster et al., 2016). Instructions were also modified to include explicit directions to rate performance with regards to the child’s or youth’s primary mode of communication (Haley et al., 2019; Kramer et al., 2012). This could have contributed to improved Social/Cognitive scores if the lack of clarity in the original version resulted in caregivers rating items without communication supports in place. Findings suggest that the PEDI-CAT (ASD) and PEDI-CAT (Original) should not be used interchangeably when assessing Social/Cognitive functioning in children and youth on the spectrum, and comparison of scores across the two versions made with appropriate caution. Moreover, careful consideration should be given to the version used when making funding decisions as the discrepancies in levels of functioning might impact on eligibility for supports, particularly if higher Social/Cognitive domain scores result in the child or youth on the spectrum being categorized as displaying functioning within the normal range (+ two *SD*; Haley et al., 2019).

Validity of the PEDI-CAT (ASD) was evaluated by determining convergent validity and content validity. Convergent validity of the PEDI-CAT (ASD) was evaluated in relation to the Vineland-3. Significant correlations were found between all PEDI-CAT (ASD) domains and the Vineland-3 core domains for both the Parent/Caregiver and Interview Forms, however no significant relationship was found between the PEDI-CAT (ASD) Responsibility domain with the Vineland-3 Motor domain for the Interview Form. Of the significant correlations, all but one was classified as poor to moderate in strength. Initially, these findings were surprising given the face validity of both measures. For example, stronger correlations might be expected between the PEDI-CAT (ASD) Social/Cognitive and Vineland-3 Communication and Socialization domains; the PEDI-CAT (ASD) Mobility and Vineland-3 Motor Skills domains; and the PEDI-CAT (ASD) Daily Activities and Responsibility domains and Vineland-3 Daily Living Skills domains. However, a previous study revealed that these seemingly comparable domains varied considerably in content when mapped against the ICF (D’Arcy et al., 2022). Furthermore, the measures collect information from different perspectives using different criteria for their rating scales; the PEDI-CAT (ASD) assesses optimal performance by rating the *difficulty* a child or youth has performing a task in a familiar context *with support*, whereas the Vineland-3 rates the *frequency* a task is performed *without support* (Haley et al., 2019; Sparrow et al., 2016). In summary, the absence of good convergent validity between the measures is likely to arise from the different conceptual frameworks on which they are based. This difference should be considered by clinicians when selecting a measure and the two measures should not be used interchangeably without consideration given to the purpose of their use.

Content validity analysis of the PEDI-CAT (ASD) revealed that it lacked comprehensiveness according to the ICF framework, covering less than half of the Autism ICF-CS, even when the entire item bank was analyzed. In practice, only selected items are administered leading to the further reduction in comprehensiveness apparent when the sample data was analyzed (the maximum coverage ranged from 26 to 30% of the age-specific Autism ICF-CS). Of the domains, Activities and Participation had the greatest coverage, with the maximum ranging from 61 to 64% of the age-specific Autism ICF-CS. A small percentage of the Body Function domain was represented, with a maximum of 6–11% coverage across the age-specific Autism ICF-CS. No codes from the Environmental Factors domain were covered, revealing a potential limitation as functioning occurs within a physical, attitudinal, and social context, and can vary markedly in different environments, particularly in relation to autism (de Schipper et al., 2016; Mahdi et al., 2018b; World Health Organization, 2001). However, the authors claim that the PEDI-CAT (ASD) was not designed to measure environmental factors separately, instead it provides relevant context within each item and captures performance in a typical daily environment with supports or modifications in place (Haley et al., 2019). Content analysis from this study confirms that the PEDI-CAT (ASD) should be considered a measure of Activities and Participation, a finding consistent with its design (Haley et al., 2019) and content analysis of the original version (D’Arcy et al., 2022; Thompson et al., 2018).

Closer scrutiny of the Activities and Participation chapters identified that the PEDI-CAT (ASD) primarily assesses Self-care, but also has a strong focus on Communication and Domestic Life (World Health Organization, 2001). While individuals on the spectrum commonly need support in these areas (Australian Bureau of Statistics, 2018c), the limited coverage of Community, Social and Civic Life, Mobility, and Major Life Areas highlights important gaps in assessment. The Autism ICF-CS contain three (second level) codes from the Community Social and Civic Life chapter, two of which were assessed by the PEDI-CAT (ASD) only once. Given that autism is characterized by differences in interpersonal interactions that impact on functioning (American Psychiatric Association, 2013), it would be expected that children and youth on the spectrum have decreased participation in social activities and are likely to experience social isolation, and research supports this (Askari et al., 2015; Orsmond et al., 2013; Shattuck et al., 2011). A recent study exploring the support needs of 68 school-aged individuals on the spectrum revealed that they experienced difficulty participating in community, social and civic life with 80% of their caregivers reporting the need for support to engage in play, hobbies, sports and informal associations (Evans et al., 2022). This may become even more apparent through the transition to adulthood, as adolescents lose the scaffolding and support provided by schools (Myers et al., 2015). Importantly, evidence shows that participation restrictions extend beyond social activities to a broad range of physical, recreational, and informal activities, highlighting the need for a comprehensive assessment to inform a participation profile (Askari et al., 2015), which also considers the individual’s interests, strengths and supports needed to enable meaningful engagement in the community and feel a sense of belonging (Askari et al., 2015; Weaver et al., 2021). Analysis of PEDI-CAT (ASD) content demonstrated that the measure lacks the ability to provide this information.

The PEDI-CAT (ASD) was found to have little coverage of the Mobility chapter; however, it is worth noting that only two second level codes (relating to using transportation and driving) are included in the Autism ICF-CS, despite the increasing evidence that many people on the spectrum experience movement difficulties (Fournier et al., 2010; Licari et al., 2020; Liu & Breslin, 2013). In Australia, 52% of individuals on the spectrum require assistance with mobility; more than a third need this support on a daily basis (Australian Bureau of Statistics, 2018c). These statistics place mobility as the second highest area of need (above self-care, communication, household chores and meal preparation; Australian Bureau of Statistics, 2018c), however this is not reflected in the Autism ICF-CS and could explain the reduced relevance of the PEDI-CAT (ASD) if it assesses mobility items (such as fine-motor skills) that aren’t recognized in the Autism ICF-CY.

The Autism ICF-CS includes eight codes from the Major Life Areas chapter, however, the only codes covered by the PEDI-CAT (ASD) were Basic economic transactions, Economic self-sufficiency, and Engagement in play. Codes relating to school and higher education, vocational training and work were not covered. According to the Australian Bureau of Statistics (2018), 93% of children and youth on the spectrum (5–20 years) attending school in Australia experience educational restrictions. Nearly 41% of these attended a non-mainstream education setting or a separate class within a mainstream school environment, and a small number were unable to attend school at all. Nearly 78% were reported to experience challenges at their educational institution, with the main difficulties associated with fitting in socially, learning, and communicating, and 46% needed more educational support (Australian Bureau of Statistics, 2018c). Individuals on the spectrum were also less likely to have post-school qualifications, with 8% holding a bachelor degree or higher, compared with 16% of individuals with a disability and 31% of individuals without a disability (Australian Bureau of Statistics, 2018c). Notwithstanding the valuable contribution individuals on the spectrum make in the workforce (Hillier et al., 2007; Hurley-Hanson et al., 2020; Jacob et al., 2015; Scott et al., 2017), only 38% of those of working age (15–64 years) in Australia were found to be employed, compared with 53% of all individuals with a disability and 84% of people without a disability (Australian Bureau of Statistics, 2018c). Moreover, nearly 90% of caregivers of school-aged children reported support needs linked to the Major Life Areas chapter (Evans et al., 2022). The transitional period to adulthood can be stressful for youth on the spectrum and their caregivers who report receiving inadequate support during this time (Evans et al., 2022; First et al., 2016; Hatfield et al., 2017). It is recommended that early and individualized transition planning occur, guided by a comprehensive assessment to develop appropriate goals and strategies (Hatfield et al., 2017, 2018; Snell-Rood et al., 2020; Test et al., 2014). However, based on the findings of the content analysis, the PEDI-CAT (ASD) has limited application in the assessment of school children and youth transitioning to post-school activities. Moreover, the PEDI-CAT (ASD) is based on caregiver-report, and it is recommended that transition planning involve the youth in the process (Chandroo et al., 2018; Hatfield et al., 2017, 2018). To address this gap, the PEDI-PRO was recently developed as a self-report measure for youth with developmental disabilities, however it requires further evaluation (Kramer & Schwartz, 2018).

Comparison of the PEDI-CAT (ASD) with the PEDI-CAT (Original) revealed similar comprehensiveness, with the PEDI-CAT (ASD) covering only one additional Autism ICF-CS code (within the Social/Cognitive domain). It is important to note that the PEDI-CATs were not designed to evaluate functioning in detail but estimate performance with greater precision and efficiency by filtering out irrelevant items based on an individual’s previous responses (Haley et al., 2019). However, this study found that only 54% of codes assigned to the PEDI-CAT (ASD) were represented in the Autism ICF-CS (a 2% improvement in relevance from the original version), suggesting that 46% of the measure assessed aspects of functioning not considered relevant to autism according to the ICF framework. Given that the PEDI-CAT (ASD) is relatively quick to administer, it is debatable whether these extra items would add considerable test burden.

Caregiver feedback on the acceptability of the PEDI-CAT (ASD) was mixed, with both positive and negative responses. The consensus was that the measure was brief and user-friendly but lacked comprehensiveness and failed to capture fluctuations in functioning experienced from day to day and across different environmental contexts, which can lead to great variability in support needs. Caregivers expressed concerns about measuring functioning with supports in situ without evaluating the nature of such supports. While the aim of the PEDI-CAT (ASD) is to use a strengths-based approach to capture the alternate ways that children and youth may *successfully* perform activities (Kramer et al., 2012), in practicality, this may lead to an overestimation of ability that precludes eligibility for funding and services to meet and maintain support needs.

A number of limitations to the research require consideration. Despite targeted recruitment, only 28 participants over 12 years of age were included in the study, and there were not enough data to analyze the 17 + years subsample. More research needs to be conducted to explore the use of the PEDI-CAT (ASD) with youth on the spectrum. Another limitation was the comprehensive assessment some families underwent in Assessment 2. The PEDI-CAT (ASD) was completed early in the assessment process minimizing the impact of fatigue on results, however, the burden of such a large assessment, together with recall bias stemming from the time lapse between Assessment 2 and 3, may have influenced caregiver feedback.

The content of a measure should address the needs of the target population (Terwee et al., 2018). Therefore, the PEDI-CAT (ASD) should be evaluated using community-based participatory research to ensure relevance (Chown et al., 2017; McConachie et al., 2015; Nicolaidis et al., 2011, 2016). This is a notable limitation of the present study; further research is needed to understand the construct of functioning and validity of the PEDI-CAT (ASD) from the perspective of individuals on the spectrum. Moreover, given that caregivers may also be on the spectrum (Xie et al., 2020), their explicit input regarding usability and accessibility should be investigated to ensure the language is accessible, clear and free from assumptions (Raymaker et al., 2017).

Overall, this study demonstrates that the PEDI-CAT (ASD) has some adequate psychometric properties as a measure of Activities and Participation and has the benefit of being user-friendly and efficient. However, it’s use requires some considerations as content analysis reveals that it lacks comprehensiveness and relevance when mapped against the Autism ICF-CS. Moreover, comparison with the PEDI-CAT (Original) and feedback from caregivers raises concerns about the potential for the PEDI-CAT (ASD) to overestimate functioning, leading to unmet support needs when used to determine eligibility for funding and services. Thus, the clinical utility of the PEDI-CAT (ASD) is limited, a finding consistent with other measures designed to assess functioning in individuals on the spectrum (D’Arcy et al., 2022; Hayden-Evans et al., 2022). It is recommended that the PEDI-CAT (ASD) be considered as one part of a comprehensive assessment of functioning and support needs, supplemented with other standardized measures (referring to D’Arcy et al. (2022) and Hayden-Evans et al. (2022) to aid selection), clinical observations, and professional consultation to collect supplementary information across various settings, including environmental facilitators and barriers (Whitehouse et al., 2018).

## Electronic supplementary material

Below is the link to the electronic supplementary material.

Supplementary Material 1

## Declarations

### Acknolwedgements

Other investigators are also acknowledged as contributing to the research design and data management, including Dr Susan Taylor, Professor Valsamma Eapen and Associate Professor John Wray. This research project would not have been possible without the involvement of our team of research assistants (Rebecca Kuzminski, Julia Tang, Rebecca Thorpe, Kerry Wallace, Dominique Cleary), research clinicians (Marie Rodatz and Melissa Scott), administrative assistants (Amy Deverell, Emma Moon and Anna Schmid), statisticians (Wesley Billingham, Matt Cooper and Richard Parsons) and volunteers (Caroline Asbury and Rebecca Vraptsis). In addition, we are grateful to the research teams and service providers who aided recruitment, along with the APM operations and assessor teams (led by Fiona Lynch) for their dedication completing Assessments 1 and 2. Finally, we would like to express our appreciation for the children, youth and caregivers who contributed their time and ideas through participating in this research project.

## References

[CR1] American Psychiatric Association. (2013). *Diagnostic and statistical manual of mental disorders: DSM-5* (5th ed.). American Psychiatric Association.

[CR2] Askari, S., Anaby, D., Bergthorson, M., Majnemer, A., Elsabbagh, M., & Zwaigenbaum, L. (2015). Participation of children and youth with autism spectrum disorder: A scoping review. *Review Journal of Autism and Developmental Disorders*, *2*(1), 103–114. 10.1007/s40489-014-0040-7.

[CR3] Australian Bureau of Statistics (2018a). *1270.0.55.005 - Australian Statistical Geography Standard (ASGS): Volume 5 - Remoteness Structure, July 2016*. Retrieved from https://www.abs.gov.au/AUSSTATS/abs@.nsf/Lookup/1270.0.55.005Main+Features1July%202016?OpenDocument.

[CR4] Australian Bureau of Statistics (2018b). *2033.0.55.001 - Census of Population and Housing: Socio-Economic Indexes for Areas (SEIFA), Australia, 2016*. Retrieved from https://www.abs.gov.au/ausstats/abs@.nsf/Lookup/by%20Subject/2033.0.55.001~2016~Main%20Features~IRSAD~20.

[CR5] Australian Bureau of Statistics (2018c). *Disability, ageing and carers, Australia: Summary of findings*. Retrieved from https://www.abs.gov.au/statistics/health/disability/disability-ageing-and-carers-australia-summary-findings/latest-release#autism-in-australia.

[CR6] Bland, J. M., & Altman, D. G. (1986). Statistical methods for assessing agreement between two methods of clinical measurement. *The Lancet*, *327*(8476), 307–310. 2868172

[CR7] Bölte, S., de Schipper, E., Robison, J. E., Wong, V. C. N., Selb, M., Singhal, N., de Vries, P. J., & Zwaigenbaum, L. (2014). Classification of functioning and impairment: The development of ICF core sets for autism spectrum disorder. *Autism Research*, *7*(1), 167–172. 10.1002/aur.1335. 24124074 10.1002/aur.1335

[CR8] Bölte, S., Mahdi, S., de Vries, P. J., Granlund, M., Robison, J. E., Shulman, C., Swedo, S., Tonge, B., Wong, V., Zwaigenbaum, L., Segerer, W., & Selb, M. (2019). The Gestalt of functioning in autism spectrum disorder: Results of the international conference to develop final consensus International Classification of Functioning, Disability and Health core sets. *Autism*, *23*(2), 449–467. 10.1177/1362361318755522. 10.1177/1362361318755522 PMC6376609 29378422

[CR9] Chandroo, R., Strnadova, I., & Cumming, T. M. (2018). A systematic review of the involvement of students with autism spectrum disorder in the transition planning process: Need for voice and empowerment. *Research in Developmental Disabilities*, *83*, 8–17. 10.1016/j.ridd.2018.07.011. 30086472 10.1016/j.ridd.2018.07.011

[CR10] Chown, N., Robinson, J., Beardon, L., Downing, J., Hughes, L., Leatherland, J., Fox, K., Hickman, L., & MacGregor, D. (2017). Improving research about us, with us: A draft framework for inclusive autism research. *Disability & Society*, *32*(5), 720–734. 10.1080/09687599.2017.1320273.

[CR11] Cicchetti, D. V. (1994). Guidelines, criteria, and rules of thumb for evaluating normed and standardised assessment instruments in psychology. *Psychological Assessment*, *6*(4), 284–290. 10.1037/1040-3590.6.4.284.

[CR12] Cieza, A., Fayed, N., Bickenbach, J., & Prodinger, B. (2019). Refinements of the ICF linking rules to strengthen their potential for establishing comparability of health information. *Disability and Rehabilitation*, *41*(5), 574–583. 10.3109/09638288.2016.1145258. 26984720 10.3109/09638288.2016.1145258

[CR13] Commonwealth of Australia (2013). *National Disability Insurance Scheme Act 2013*. Retrieved from https://www.legislation.gov.au/Details/C2013A00020.

[CR14] Coster, W. J., Kramer, J. M., Tian, F., Dooley, M., Liljenquist, K., Kao, Y. C., & Ni, P. (2016). Evaluating the appropriateness of a new computer-administered measure of adaptive function for children and youth with autism spectrum disorders. *Autism*, *20*(1), 14–25. 10.1177/1362361314564473. 25630376 10.1177/1362361314564473 PMC4661128

[CR15] D’Arcy, E., Wallace, K., Chamberlain, A., Evans, K., Milbourn, B., Bölte, S., Whitehouse, A. J. O., & Girdler, S. (2022). Content validation of common measures of functioning for young children against the International Classification of Functioning, disability and health and code and core sets relevant to neurodevelopmental conditions. *Autism*, *26*(4), 928–939. 10.1177/13623613211036809. 34369196 10.1177/13623613211036809 PMC9008546

[CR16] D’Arcy, E., Evans, K., Afsharnejad, B., Milbourn, B., Bölte, S., & Girdler, S. (2023). Assessing functioning for individuals with neurodevelopmental conditions: Current clinical practice in Australia. *Australian Journal of Occupational Therapy*, *70*(1), 43–60. 10.1111/1440-1630.12834. 10.1111/1440-1630.12834 PMC10087051 35934786

[CR17] de Schipper, E., Mahdi, S., de Vries, P., Granlund, M., Holtmann, M., Karande, S., Almodayfer, O., Shulman, C., Tonge, B., Wong, V. C. N., Zwaigenbaum, L., & Bölte, S. (2016). Functioning and disability in autism spectrum disorder: A worldwide survey of experts. *Autism Research*, *9*(9), 959–969. 10.1002/aur.1592. 26749373 10.1002/aur.1592 PMC5064728

[CR18] DePoy, E., & Gitlin, L. N. (2015). *Introduction to research-e-book: Understanding and applying multiple strategies*. Elsevier Health Sciences.

[CR19] Dunn, T. J., Baguley, T., & Brunsden, V. (2014). From alpha to omega: A practical solution to the pervasive problem of internal consistency estimation. *British Journal of Psychology*, *105*(3), 399–412. 10.1111/bjop.12046. 24844115 10.1111/bjop.12046

[CR20] Evans, K., Whitehouse, A. J. O., D’Arcy, E., Hayden-Evans, M., Wallace, K., Kuzminski, R., Thorpe, R., Girdler, S., Milbourn, B., Bölte, S., & Chamberlain, A. (2022). Perceived support needs of school-aged young people on the autism spectrum and their caregivers. *International Journal of Environmental Research and Public Health*, *19*(23), 15605. 10.3390/ijerph192315605. 36497683 10.3390/ijerph192315605 PMC9737194

[CR21] First, J., Cheak-Zamora, N. C., & Teti, M. (2016). A qualitative study of stress and coping when transitioning to adulthood with autism spectrum disorder. *Journal of Family Social Work*, *19*(3), 220–236. 10.1080/10522158.2016.1185074.

[CR22] Foster, M., Henman, P., Tilse, C., Fleming, J., Allen, S., & Harrington, R. (2016). Reasonable and necessary’care: The challenge of operationalising the NDIS policy principle in allocating disability care in Australia. *Australian Journal of Social Issues*, *51*(1), 27–46. 10.1002/j.1839-4655.2016.tb00363.x.

[CR23] Fournier, K. A., Hass, C. J., Naik, S. K., Lodha, N., & Cauraugh, J. H. (2010). Motor coordination in autism spectrum disorders: A synthesis and meta-analysis. *Journal of Autism and Developmental Disorders*, *40*(10), 1227–1240. 10.1007/s10803-010-0981-3. 20195737 10.1007/s10803-010-0981-3

[CR24] Gleason, K., & Coster, W. (2012). An ICF-CY-based content analysis of the Vineland Adaptive Behavior Scales-II. *Journal of Intellectual and Developmental Disability*, *37*(4), 285–293. 10.3109/13668250.2012.720675. 22989139 10.3109/13668250.2012.720675

[CR25] Haley, S. M., Coster, W. J., Dumas, H. M., Fragala-Pinkham, M. A., Moed, R., Kramer, J., Ni, P., Feng, T., Kao, Y. C., & Ludlow, L. H. (2019). *Pediatric Evaluation of Disability Inventory: Development, standardization and administration manual (Version 1.4.3)*. Boston University.

[CR26] Harris, P., Taylor, R., Thielke, R., Payne, J., Gonzalez, N., & Conde, J. (2009). Research electronic data capture (REDCap) – a metadata-driven methodology and workflow process for providing translational research informatics support. *Journal of Biomedical Informatics*, *42*(2), 377–381. 18929686 10.1016/j.jbi.2008.08.010 PMC2700030

[CR27] Harris, P., Taylor, R., Minor, B., Elliott, V., Fernandez, M., O’Neal, L., McLeod, L., Delacqua, G., Delacqua, F., Kirby, J., & Dudaacon, S. N. (2019). The REDCap consortium: Building an international community of software partners. *Journal of Biomedical Informatics*, *95*, 103208. 10.1016/j.jbi.2019.103208. 31078660 10.1016/j.jbi.2019.103208 PMC7254481

[CR28] Harrison, P., & Oakland, T. (2015). *Adaptive behavior assessment system* (3rd ed.). Western Psychological Services.

[CR29] Hatfield, M., Falkmer, M., Falkmer, T., & Ciccarelli, M. (2017). Leaps of faith: Parents’ and professionals’ viewpoints on preparing adolescents on the autism spectrum for leaving school. *Journal of Research in Special Educational Needs*, *17*(3), 187–197. 10.1111/1471-3802.12377.

[CR30] Hatfield, M., Ciccarelli, M., Falkmer, T., & Falkmer, M. (2018). Factors related to successful transition planning for adolescents on the autism spectrum. *Journal of Research in Special Educational Needs*, *18*(1), 3–14. 10.1111/1471-3802.12388.

[CR31] Hayden-Evans, M., Milbourn, B., D’Arcy, E., Chamberlain, A., Afsharnejad, B., Evans, K., Whitehouse, A. J. O., Bölte, S., & Girdler, S. (2022). An evaluation of the overall utility of measures of functioning suitable for school-aged children on the autism spectrum: A scoping review. *International Journal of Environmental Research and Public Health*, *19*(21), 14114. 10.3390/ijerph192114114. 36360993 10.3390/ijerph192114114 PMC9659140

[CR32] Hillier, A., Campbell, H., Mastriani, K., Izzo, M., Kool-Tucker, A., & Beversdorf, D. (2007). Two-year evaluation of a vocational support program for adults on the autism spectrum. *Career Development and Transition for Exceptional Individuals*, *30*(1), 125–134.

[CR33] Hurley-Hanson, A. E., Giannantonio, C. M., & Griffiths, A. J. (2020). The benefits of employing individuals with autism. In A. E. Hurley-Hanson, C. M. Giannantonio, & A. J. Griffiths (Eds.), *Autism in the workplace creating positive employment and career outcomes for Generation A*. Palgrave Macmillan. 10.1007/978-3-030-29049-8_12.

[CR34] IBM Corp (2020). *IBM SPSS Statistics for Windows*. In (Version 27.0) [Computer software]. IBM Corp. https://www.ibm.com/au-en/products/spss-statistics?mhsrc=ibmsearch_a&mhq=spss

[CR35] Jacob, A., Scott, M., Falkmer, M., & Falkmer, T. (2015). The costs and benefits of employing an adult with autism spectrum disorder: A systematic review. *Plos One*, *10*(10), e0139896. 10.1371/journal.pone.0139896. 26445345 10.1371/journal.pone.0139896 PMC4596848

[CR36] Koo, T. K., & Li, M. Y. (2016). A guideline of selecting and reporting intraclass correlation coefficients for reliability research. *Journal of Chiropractic Medicine*, *15*(2), 155–163. 10.1016/j.jcm.2016.02.012. 27330520 10.1016/j.jcm.2016.02.012 PMC4913118

[CR37] Kramer, J. M., & Schwartz, A. E. (2018). Development of the Pediatric disability inventory-patient reported Outcome (PEDI-PRO) measurement conceptual framework and item candidates. *Scandinavian Journal of Occupational Therapy*, *25*(5), 335–346. 10.1080/11038128.2018.1502344. 30280615 10.1080/11038128.2018.1502344 PMC6377807

[CR38] Kramer, J. M., Coster, W. J., Kao, Y. C., Snow, A., & Orsmond, G. I. (2012). A new approach to the measurement of adaptive behavior: Development of the PEDI-CAT for children and youth with autism spectrum disorders. *Physical and Occupational Therapy in Pediatrics*, *32*(1), 34–47. 10.3109/01942638.2011.606260. 21846290 10.3109/01942638.2011.606260 PMC3272487

[CR39] Kramer, J. M., Liljenquist, K., P., N., & Coster, W. (2015). Examining differential responses of youth with and without autism on a measure of everyday activity performance. *Quality of Life Research*, *24*(12), 2993–3000. 10.1007/s11136-015-1035-2. 26063170 10.1007/s11136-015-1035-2 PMC4619133

[CR40] Kramer, J. M., Liljenquist, K., & Coster, W. J. (2016). Validity, reliability, and usability of the Pediatric evaluation of disability inventory-computer adaptive test for autism spectrum disorders. *Developmental Medicine and Child Neurology*, *58*(3), 255–261. 10.1111/dmcn.12837. 26104112 10.1111/dmcn.12837 PMC4688240

[CR41] Licari, M. K., Alvares, G. A., Varcin, K., Evans, K. L., Cleary, D., Reid, S. L., Glasson, E. J., Bebbington, K., Reynolds, J. E., Wray, J., & Whitehouse, A. J. O. (2020). Prevalence of motor difficulties in autism spectrum disorder: Analysis of a population-based cohort. *Autism Research*, *13*, 198–306. 10.1002/aur.2230. 10.1002/aur.2230 31625694

[CR42] Liu, T., & Breslin, C. M. (2013). Fine and gross motor performance of the MABC-2 by children with autism spectrum disorder and typically developing children. *Research in Autism Spectrum Disorders*, *7*(10), 1244–1249. 10.1016/j.rasd.2013.07.002.

[CR43] Mahdi, S., Albertowski, K., Almodayfer, O., Arsenopoulou, V., Carucci, S., Dias, J. C., Khalil, M., Knüppel, A., Langmann, A., & Lauritsen, M. B. (2018a). An international clinical study of ability and disability in autism spectrum disorder using the WHO-ICF framework. *Journal of Autism and Developmental Disorders*, *48*(6), 2148–2163. https://doi.org/0.1007/s10803-018-3482-4. 10.1007/s10803-018-3482-4 PMC5948258 29423605

[CR44] Mahdi, S., Viljoen, M., Yee, T., Selb, M., Singhal, N., Almodayfer, O., Granlund, M., de Vries, P. J., Zwaigenbaum, L., & Bölte, S. (2018b). An international qualitative study of functioning in autism spectrum disorder using the World Health Organization international classification of functioning, disability and health framework. *Autism Research*, *11*(3), 463–475. 10.1002/aur.1905. 29226604 10.1002/aur.1905 PMC5900830

[CR45] McConachie, H., Parr, J. R., Glod, M., Hanratty, J., Livingstone, N., Oono, I. P., Robalino, S., Baird, G., Beresford, B., & Charman, T. (2015). Systematic review of tools to measure outcomes for young children with autism spectrum disorder. *Health Technology Assessment*, *19*(41), 1–538. 10.3310/hta19410. 26065374 10.3310/hta19410 PMC4781156

[CR46] McDonald, R. (1999). *Test theory: A unified treatment (1st ed.)*. Psychology Press. 10.4324/9781410601087.

[CR47] Myers, E., Davis, B., Stobbe, G., & Bjornson, K. (2015). Community and social participation among individuals with autism spectrum disorder transitioning to adulthood. *Journal of Autism and Developmental Disorders*, *45*(8), 2373–2381. 10.1007/s10803-015-2403-z. 25725812 10.1007/s10803-015-2403-z

[CR48] National Disability Insurance Agency (2019). *Providing evidence of disability for children* Applying to access the NDIS. https://www.ndis.gov.au/applying-access-ndis/how-apply/information-support-your-request/providing-evidence-disability-children.

[CR49] National Health and Medical Research Council, Australian Research Council, & Universities Australia (2018a). *Australian Code for the Responsible Conduct of Research*. https://www.nhmrc.gov.au/about-us/publications/australian-code-responsible-conduct-research-2018.

[CR50] National Health and Medical Research Council, Australian Research Council, & Universities Australia (2018b). *The National Statement on Ethical Conduct in Human Research 2007 (Updated 2018)*. https://www.nhmrc.gov.au/about-us/publications/national-statement-ethical-conduct-human-research-2007-updated-2018#block-views-block-file-attachments-content-block-1.

[CR51] National Institute for Health and Care Excellence (2013). *Autism spectrum disorder in under 19s: Support and management*. Retrieved from https://www.nice.org.uk/guidance/cg170. 34283415

[CR52] Nicolaidis, C., Raymaker, D., McDonald, K., Dern, S., Ashkenazy, E., Boisclair, C., Robertson, S., & Baggs, A. (2011). Collaboration strategies in nontraditional community-based participatory research partnerships: Lessons from an academic–community partnership with autistic self-advocates. *Progress in Community Health Partnerships*, *5*(2), 143–150. 10.1353/cpr.2011.0022. 21623016 10.1353/cpr.2011.0022 PMC3319698

[CR53] Nicolaidis, C., Raymaker, D., McDonald, K., Kapp, S., Weiner, M., Ashkenazy, E., Gerrity, M., Kripke, C., Platt, L., & Baggs, A. (2016). The development and evaluation of an online healthcare toolkit for autistic adults and their primary care providers. *Journal of General Internal Medicine*, *31*(10), 1180–1189. 10.1007/s11606-016-3763-6. 27271730 10.1007/s11606-016-3763-6 PMC5023610

[CR54] Orsmond, G., Shattuck, P., Cooper, B., Sterzing, P., & Anderson, K. (2013). Social participation among young adults with an autism spectrum disorder. *Journal of Autism and Developmental Disorders*, *43*, 2710–2719. 10.1007/s10803-013-1833-8. 23615687 10.1007/s10803-013-1833-8 PMC3795788

[CR55] Pearson Clinical Assessment (2022). *Q-Global*. https://www.pearsonclinical.com.au/products/view/507.

[CR56] Portney, L. G., & Watkins, M. P. (2009). *Foundations of clinical research: Applications to practice* (3rd ed.).). Pearson/Prentice Hall.

[CR57] QSR International Pty Ltd (2020). *NVivo*. https://www.qsrinternational.com/nvivo-qualitative-data-analysis-software/home.

[CR58] Raymaker, D. M., McDonald, K. E., Ashkenazy, E., Gerrity, M., Baggs, A. M., Kripke, C., Hourston, S., & Nicolaidis, C. (2017). Barriers to healthcare: Instrument development and comparison between autistic adults and adults with and without other disabilities. *Autism*, *21*(8), 972–984. 10.1177/1362361316661261. 27663266 10.1177/1362361316661261 PMC5362353

[CR59] Scott, M., Jacob, A., Hendrie, D., Parsons, R., Girdler, S., Falkmer, T., & Falkmer, M. (2017). Employers’ perception of the costs and the benefits of hiring individuals with autism spectrum disorder in open employment in Australia. *Plos One*, *12*(5), e0177607. 10.1371/journal.pone.0177607. 28542465 10.1371/journal.pone.0177607 PMC5436808

[CR60] Shattuck, P. T., Orsmond, G. I., Wagner, M., & Cooper, B. P. (2011). Participation in social activities among adolescents with an autism spectrum disorder. *Plos One*, *6*(11). 10.1371/journal.pone.0027176. Article e27176. 10.1371/journal.pone.0027176 PMC3215697 22110612

[CR61] Snell-Rood, C., Ruble, L., Kleinert, H., McGrew, J. H., Adams, M., Rodgers, A., Odom, J., Wong, W. H., & Yu, Y. (2020). Stakeholder perspectives on transition planning, implementation, and outcomes for students with autism spectrum disorder. *Autism*, *24*(5), 1164–1176. 10.1177/1362361319894827. 31957461 10.1177/1362361319894827 PMC7311242

[CR62] Sparrow, S. S., Cicchett, D., & Balla, D. A. (2005). *Vineland Adaptive Behavior scales, Second Edition (Vineland-II)*. American Guidance Service.

[CR63] Sparrow, S., Cicchetti, D., & Saulnier, C. (2016). *Vineland Adaptive Behaviour Scales, Third Edition*. Pearson Inc.

[CR64] Terwee, C. B., Prinsen, C. A. C., Chiarotto, A., de Vet, H. C. W., Bouter, L. M., Alonso, J., Westerman, M. J., Patrick, D. L., & Mokkink, L. B. (2018). *COSMIN methodology for assessing the content validity of PROMs-user manual*. VU University Medical Center. 10.1007/s11136-018-1829-0 PMC5891557 29550964

[CR65] Test, D. W., Smith, L. E., & Carter, E. W. (2014). Equipping youth with autism spectrum disorders for adulthood: Promoting rigor, relevance, and relationships. *Remedial and Special Education*, *35*(2), 80–90. 10.1177/0741932513514857.

[CR66] The R Foundation (n.d.). *What is R?* https://www.r-project.org/about.html.

[CR67] Thompson, S. V., Cech, D. J., Cahill, S. M., & Krzak, J. J. (2018). Linking the Pediatric evaluation of disability inventory-computer adaptive test (PEDI-CAT) to the International classification of function. *Pediatric Physical Therapy*, *30*(2), 113–118. 10.1097/PEP.0000000000000483. 29498960 10.1097/PEP.0000000000000483

[CR68] Weaver, L. A., Bingham, E., Luo, K., Juárez, A. P., & Taylor, J. L. (2021). What do we really mean by inclusion? The importance of terminology when discussing approaches to community engagement. *Autism*, *25*(8), 2149–2151. 10.1177/136236132110466. 34519551 10.1177/13623613211046688

[CR69] Whitehouse, A. J. O., Evans, K., Eapen, V., & Wray, J. (2018). *A national guideline for the assessment and diagnosis of autism spectrum disorders in Australia. Summary and recommendations*. Cooperative Research Centre for Living with Autism.

[CR70] World Health Organization (2001). *International Classification of Functioning, Disability and Health (ICF)*. https://www.who.int/standards/classifications/international-classification-of-functioning-disability-and-health.

[CR71] Xie, S., Karlsson, H., Dalman, C., Widman, L., Rai, D., Gardner, R. M., Magnusson, C., Sandin, S., Tabb, L. P., Newschaffer, C. J., & Lee, B. K. (2020). The familial risk of autism spectrum disorder with and without intellectual disability. *Autism Research*, *13*(12), 2242–2250. 10.1002/aur.2417. 33103358 10.1002/aur.2417 PMC7821228

