# Supplementary material for: Reliability, Validity and Acceptability of the PEDI-CAT with ASD Scales for Australian Children and Youth on the Autism Spectrum
Source: J Autism Dev Disord. 2024 Apr 28;55(7):2422–35. doi: 10.1007/s10803-024-06366-7 (PMC12167268; doi:10.1007/s10803-024-06366-7)
Supplement: Supplementary file 1 — Supplementary Material 1 [file 10803_2024_6366_MOESM1_ESM.pdf]

Supplementary File S1

Reliability, Validity and Acceptability of the PEDI-CAT with ASD Scales for Australian Children and Youth on the Autism Spectrum

Journal of Autism and Developmental Disorders https://doi.org/10.1007/s10803-024-06366-7

Angela Chamberlain[1,2,3], Maya Hayden-Evans[1,2,3], Emily D'Arcy[1,2,3], Andrew JO Whitehouse[2,3,6], Kerry Wallace[2], Sonya Girdler[1,2,3,4,5], Benjamin Milbourn[1,2,3], Sven Bölte[1,4,6], and Kiah Evans[1,2,3,5].

Corresponding Author: kiah.evans@uwa.edu.au

[1] Curtin University, Perth, Australia

[2] Telethon Kids Institute, Perth, Australia

[3] Cooperative Research Centre for Living with Autism, Brisbane, Australia

[4] Karolinska Institutet, Solna, Sweden

[5] The University of Western Australia, Perth, Australia

[6] Stockholm Health Care Services, Stockholm, Sweden

Table 1

Caregiver Interview Questions

Question
The results from this assessment found [summary of findings].<br>1. What are your thoughts on hearing those results?<br>2. How well do you think this assessment tool accurately measured [name of child or youth]'s actual challenges and abilities in everyday life?<br>3. What did you like about this assessment tool?<br>4. What did you not like about this assessment tool?<br>5. Are there any improvements you can suggest for this assessment tool?

Table 2

Characteristics of the Child or Youth and their Caregiver(s)

Characteristic	Child or youth<br>*n* = 134(%)	Caregiver[a]<br>*n* = 130(%)
Relationship to child or youth		
Biological mother		118(90.8)
Biological father		7(5.4)
Other		3(2.3)
Missing		2(1.5)
Gender		
Female	38(28.4)	12(93.1)
Male	96(71.6)	7(5.4)
Missing	0	2(1.5)
Identifies with specific cultural group		
Yes	18(13.4)	22(16.9)
No	110(82.1)	104(80.0)
Missing	6(4.5)	4(3.1)
Speaks a language other than English at home		
Yes	7(5.2)	28(21.5)
No	121(90.3)	98(75.4)
Missing	6(4.5)	4(3.1)
Presence of co-occurring condition		
Yes	80(59.7)	
No	54(40.3)	

https://doi.org/10.1007/s10803-024-06366-7

Co-occurring intellectual disability			
Yes	31(23.1)		
No	103(76.9)		
Co-occurring global developmental delay			
Yes	16(11.9)		
No	118(88.1)		
Co-occurring motor disorder			
Yes	13(9.7)		
No	121(90.3)		
Co-occurring communication disorder			
Yes	29(21.6)		
No	105(78.4)		
Co-occurring attention deficit hyperactivity disorder			
Yes	43(32.1)		
No	91(67.9)		
Geographical location			
Major city		118(90.8)	
Inner regional		8(6.2)	
Outer regional		4(3.1)	
SEIFA IRSAD			
Median		8	
Range		1-10	
Age in years			
Mean(SD)	9.53(3.7)		42.02(7.26)[b]
Range	3.48 – 18.93		26.61 – 67.24

Note. SEIFA = Socio-Economic Indexes for Areas; IRSAD = Index of Relative Socio-Economic Advantage and Disadvantage.
[a]The number of caregivers is less than children and youth, as four families had two children participate in the study.
[b]*n* = 124.

Table 3

DSM-5 Severity Levels of the Child or Youth

DSM-5 Severity Levels	*n* = 108(%)
Social Communication	
Level 1 (Requiring support)	40(37.0)
Level 2 (Requiring substantial support)	49(45.4)
Level 3 (Requiring very substantial support)	19(17.6)
Restricted, Repetitive Behaviours	
Level 1 (Requiring support)	45(41.7)
Level 2 (Requiring substantial support)	53(49.1)
Level 3 (Requiring very substantial support)	10(9.2)

https://doi.org/10.1007/s10803-024-06366-7

Figure 1

Bland-Altman Plots Showing the Difference in Mean Scores for the PEDI-CAT (ASD) and PEDI-CAT (Original) Domains: a) Daily Activities; b) Mobility; c) Social / Cognitive; and d) Responsibility

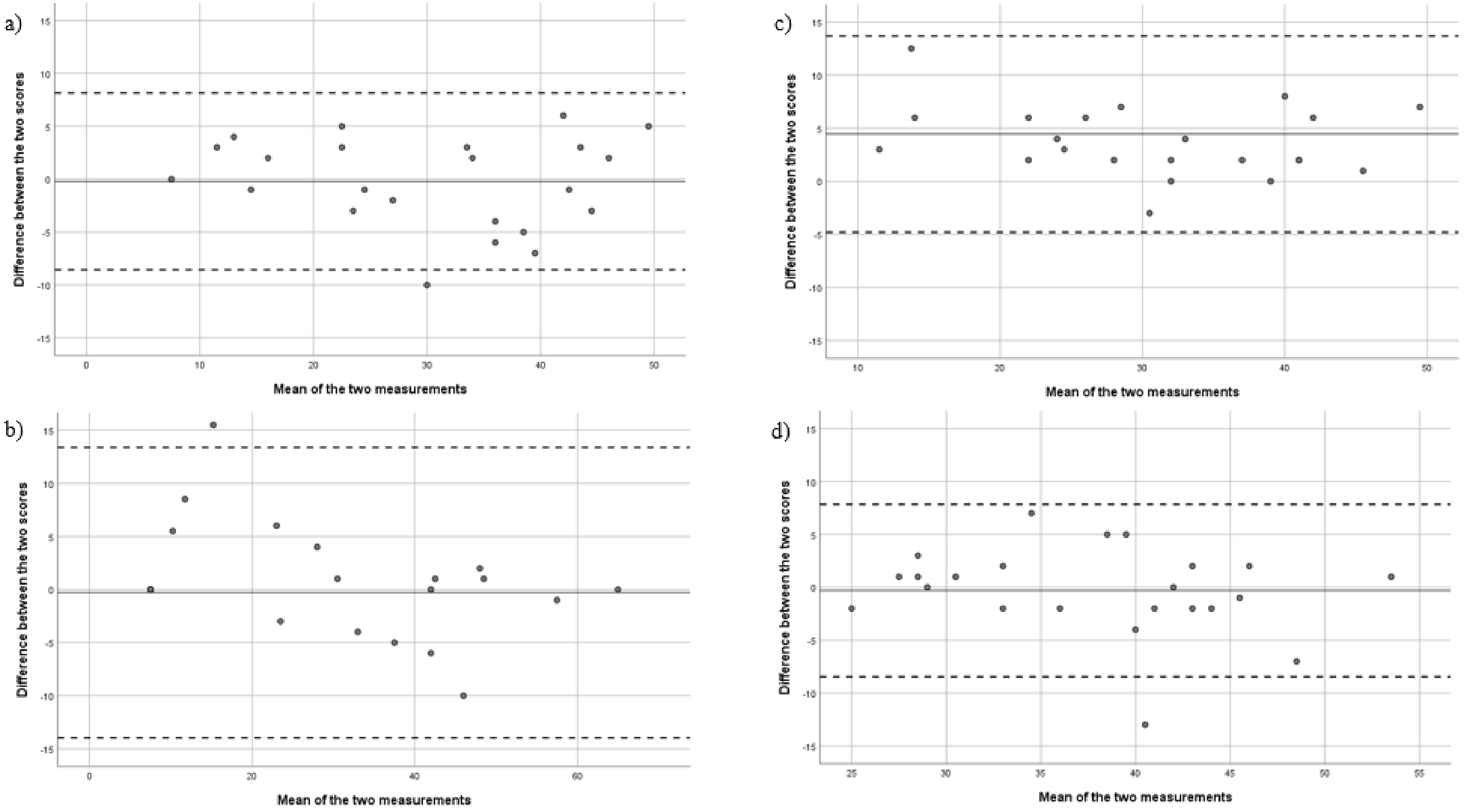

https://doi.org/10.1007/s10803-024-06366-7

Table 4

Percentage of the ICF Core Set for Autism Covered by the PEDI-CAT (ASD) and PEDI-CAT (Original) Full Item Banks

	PEDI-CAT (ASD) domains Number of codes (%)						PEDI-CAT (Original) domains Number of codes (%)					
	Full item bank	Daily Activities	Mobility	Mobility (device)	Social/Cognitive	Responsibility	Full item bank	Daily Activities	Mobility	Mobility (device)	Social/Cognitive	Responsibility
Full Core Set	46(42)	10(9)	3(3)	1(1)	26(24)	21(19)	45(41)	10(9)	3(3)	1(1)	25(23)	21(19)
Body Functions	2(10)	0(0)	0(0)	0(0)	2(10)	1(5)	2(10)	0(0)	0(0)	0(0)	2(10)	1(5)
Activity and Participation	44(75)	10(17)	3(5)	1(2)	24(41)	20(34)	43(73)	10(17)	3(5)	1(2)	23(39)	20(34)
Environmental Factors	0(0)	0(0)	0(0)	0(0)	0(0)	0(0)	0(0)	0(0)	0(0)	0(0)	0(0)	0(0)

https://doi.org/10.1007/s10803-024-06366-7

Table 5

ICF Core Set for Autism Codes Covered by the PEDI-CAT (ASD) and PEDI-CAT (Original)

ICF chapter and code	PEDI-CAT (ASD)	PEDI-CAT (Original)
Body Functions		
b1 Mental Functions		
b114 Orientation functions	0	0
b117 Intellectual functions	0	0
b122 Global psychosocial functions	0	0
b125 Dispositions and intra-personal functions	0	0
b126 Temperament and personality functions	0	0
b130 Energy and drive functions (G)	0	0
b134 Sleep functions	0	0
b140 Attention functions	2	1
b144 Memory functions	0	0
b147 Psychomotor functions	0	0
b152 Emotional functions (G)	0	0
b156 Perceptual functions	0	0
b160 Thought functions	0	0
b164 Higher-level cognitive functions	13	12
b167 Mental functions of language	0	0
b2 Sensory functions and pain		
b265 Touch function	0	0
b270 Sensory functions related to temperature and other stimuli	0	0
b3 Voice and speech functions		
b330 Fluency and rhythm of speech functions	0	0
b4 Neuromusculoskeletal and movement-related functions		
b760 Control of voluntary movement functions	0	0
b765 Involuntary movement functions	0	0
Activities and Participation		
d1 Learning and applying knowledge		
d110 Watching	1	1
d115 Listening	2	2
d130 Copying	0	0
d132 Acquiring information	1	1
d137 Acquiring concepts	1	1
d140 Learning to read	2	2
d145 Learning to write	1	1
d155 Acquiring skills	0	0
d160 Focusing attention	0	0
d161 Directing attention	0	0

https://doi.org/10.1007/s10803-024-06366-7

d163 Thinking	1	1
d166 Reading	1	1
d170 Writing	3	3
d175 Solving problems	1	1
d177 Making decisions	0	0
d2 General tasks and demands		
d210 Undertaking a single task	0	0
d220 Undertaking multiple tasks	2	1
d230 Carrying out daily routine (G)	6	6
d240 Handling stress and other psychological demands	3	2
d250 Managing one's own behaviour	6	4
d3 Communication		
d310 Communicating with - receiving - spoken messages	1	1
d315 Communicating with - receiving - nonverbal messages	2	2
d330 Speaking	7	6
d331 Pre-talking	1	0
d335 Producing nonverbal messages	5	2
d350 Conversation	4	3
d360 Using communication devices and techniques	2	2
d4 Mobility		
d470 Using transportation	4	4
d475 Driving	2	2
d5 Self-care		
d510 Washing oneself	8	6
d520 Caring for body parts	10	8
d530 Toileting	5	5
d540 Dressing	19	18
d550 Eating	7	6
d570 Looking after one's health	9	9
d571 Looking after one's safety	14	11
d6 Domestic Life		
d620 Acquisition of goods and services	2	2
d630 Preparing meals	7	8
d640 Doing housework	9	8
d650 Caring for household objects	2	2
d660 Assisting others	1	1
d7 Interpersonal interactions and relationships		
d710 Basic interpersonal interactions	6	6
d720 Complex interpersonal interactions	4	3
d730 Relating with strangers	0	0
d740 Formal relationships	1	1
d750 Informal social relationships	1	1
d760 Family relationships	0	0
d770 Intimate relationships	0	0

https://doi.org/10.1007/s10803-024-06366-7

d8 Major life areas		
d820 School education	0	0
d825 Vocational training	0	0
d830 Higher education	0	0
d845 Acquiring, keeping and terminating a job	0	0
d850 Remunerative employment (G)	0	0
d860 Basic economic transactions	1	1
d870 Economic self-sufficiency	2	2
d880 Engagement in play	5	4
d9 Community, social and civic life		
d910 Community life	1	1
d920 Recreation and leisure	1	1
d940 Human rights	0	0
Environmental Factors		
e1 Products and technology		
e110 Products or substances for personal consumption	0	0
e115 Products and technology for personal use in daily living	0	0
e125 Products and technology for communication	0	0
e130 Products and technology for education	0	0
e2 Natural environment and human-made changes to environment		
e240 Light	0	0
e250 Sound	0	0
e3 Support and relationships		
e310 Immediate family	0	0
e315 Extended family	0	0
e320 Friends	0	0
e325 Acquaintances, peers, colleagues, neighbours and community members	0	0
e330 People in positions of authority	0	0
e340 Personal care providers and personal assistants	0	0
e355 Health professionals	0	0
e360 Other professionals	0	0
e4 Attitudes		
e410 Individual attitudes of immediate family members	0	0
e415 Individual attitudes of extended family members	0	0
e420 Individual attitudes of friends	0	0
e430 Individual attitudes of people in positions of authority	0	0
e450 Individual attitudes of health professionals	0	0
e455 Individual attitudes of other professionals	0	0
e460 Societal attitudes	0	0
e465 Social norms, practices and ideologies	0	0
e5 Services, systems and policies		
e525 Housing services, systems and policies	0	0
e535 Communication services, systems and policies	0	0

https://doi.org/10.1007/s10803-024-06366-7

e550 Legal services, systems and policies	0	0
e560 Media services, systems and policies	0	0
e570 Social security services, systems and policies	0	0
e575 General social support services, systems and policies	0	0
e580 Health services, systems and policies	0	0
e585 Education and training services, systems and policies	0	0
e590 Labour and employment services, systems and policies	0	0

https://doi.org/10.1007/s10803-024-06366-7

Table 6

Percentage of the PEDI-CAT (ASD) and PEDI-CAT (Original) Relevant to the ICF Core Set for Autism (Using the Full Bank of 276 PEDI-CAT Items)

	PEDI-CAT (ASD) domains Number of codes (%total)						PEDI-CAT (Original) domains Number of codes (%total)					
	Full item bank	Daily Activities	Mobility	Mobility (device)	Social/Cognitive	Responsibility	Full item bank	Daily Activities	Mobility	Mobility (device)	Social/Cognitive	Responsibility
Full Core Set	189(54)	53(71)	6(6)	1(3)	68(81)	61(98)	166(52)	48(71)	6(6)	1(3)	57(83)	54(98)
Body Functions	15(79)	0(0)	0(0)	0(0)	7(70)	8(100)	13(76)	0(0)	0(0)	0(0)	6(67)	7(100)
Activity and Participation	174(53)	53(72)	6(6)	1(3)	61(82)	53(98)	153(50)	48(72)	6(6)	1(3)	51(85)	47(98)
Environmental Factors	0(0)	0(0)	0(0)	0(0)	0(0)	0(0)	0(0)	0(0)	0(0)	0(0)	0(0)	0(0)

https://doi.org/10.1007/s10803-024-06366-7

Table 7

Percentage of the ICF Core Set for Autism for 0–5-Year-Olds Covered by the PEDI-CAT (ASD) Items Administered to 0-5 Years Subsample (n = 26)

	Autism ICF-CS Number of codes (%)			Autism 0-5 ICF-CS Number of codes (%)		
	Median	Minimum	Maximum	Median	Minimum	Maximum
Full Core Set	30(27)	28(25)	32(29)	17(23)	16(22)	19(26)
Body Functions	1(5)	1(5)	2(10)	1(5)	1(5)	2(11)
Activity and Participation	29(49)	27(46)	31(53)	16(55)	15(52)	18(62)
Environmental Factors	0(0)	0(0)	0(0)	0(0)	0(0)	0(0)

Table 8

Percentage of the ICF Core Set for Autism for 6–16-Year-Olds Covered by the PEDI-CAT (ASD) Items Administered to 6-11 Years Subsample (n = 57)

	Autism ICF-CS Number of codes (%)			Autism 0-5 ICF-CS Number of codes (%)		
	Median	Minimum	Maximum	Median	Minimum	Maximum
Full Core Set	31(28)	25(23)	35(32)	21(26)	18(22)	24(30)
Body Functions	1(5)	1(5)	1(5)	1(6)	1(6)	1(6)
Activity and Participation	30(51)	24(41)	34(58)	20(56)	17(47)	23(64)
Environmental Factors	0(0)	0(0)	0(0)	0(0)	0(0)	0(0)

https://doi.org/10.1007/s10803-024-06366-7

Table 9

Percentage of the ICF Core Set for Autism for 6–16-Year-Olds Covered by the PEDI-CAT (ASD) Items Administered to 12-16 Years Subsample (n = 28)

	Autism ICF-CS Number of codes (%)			Autism 0-5 ICF-CS Number of codes (%)		
	Median	Minimum	Maximum	Median	Minimum	Maximum
Full Core Set	32(29)	27(25)	36(33)	21(26)	19(23)	23(28)
Body Functions	1(5)	1(5)	2(10)	1(6)	1(6)	2(11)
Activity and Participation	31(53)	25(42)	35(59)	20(56)	18(50)	22(61)
Environmental Factors	0(0)	0(0)	0(0)	0(0)	0(0)	0(0)

https://doi.org/10.1007/s10803-024-06366-7

Acceptability Results

Feedback on the acceptability of the PEDI-CAT (ASD) was provided by 43 caregivers via interview (n = 20) and/or online survey (n = 23). Two key themes emerged:

Accuracy

Many of the caregivers reported that the PEDI-CAT (ASD) accurately measured their child's or youth's abilities and challenges in everyday life. These sentiments were captured in phrases such as: "I agree with these", "spot on", "similar to what I expected", "I don't feel surprised by the results", and "pretty standard when compared to usual assessments". Caregivers reasoned that the accuracy of the PEDI-CAT (ASD) reflected its detailed nature, ease of use and relevance of the questions.

In contrast, some caregivers reported that the results did not accurately measure their child's or youth's level of functioning. Several explicitly stated the score was below expected, while others asserted the results overestimated performance. Several reasons were given for this, including assessment items that were not deemed age appropriate or relevant to autism (especially with young people who are minimally-speaking, capable but choose not to complete tasks, and/or engage in masking behaviours): "Physically [he] doesn't have an issue. Whether he does them is [what] the question isn't measuring". In addition, some caregivers felt the response categories were too broad and the measure "needs more sensitive scales" to better discriminate abilities from challenges.

Some caregivers felt the assessment did not comprehensively measure all aspects of functioning, such as strengths; the impact of sensory, emotional and psychological challenges on

https://doi.org/10.1007/s10803-024-06366-7

performance; fluctuations in functioning (across temporal and physical environments); and level of support required for success. For example:

"Because [my child]'s challenges are not physical, I feel the PEDI-CAT doesn't record accurately. [My child]'s results can vary day to day more dependent on mental/psychological challenges."

"With the social/cognitive part, [the PEDI-CAT (ASD)] doesn't consider doing something in a peer group vs individually."

"Level of behaviour/functioning for all domains showed as within expected range for age. I feel this is not a true reflection. I provide a lot of additional supports for [my child] and I can compare this to the supports I provide to my younger 3-year-old neurotypical boy. [My child's] behaviour/functioning needs assistance across all of the assessed domains. We constantly provide consistency in routine and use visuals for changes. My child requires constant reminders with high parental support to achieve daily activities."

Usefulness

Some caregivers commented that the PEDI-CAT (ASD) was useful in that it confirmed or increased their understanding of their child's or youth's functioning; provided an indicator of typical expectations; and provided information helpful in review meetings and support planning: "The results for the sections would be useful to support us if we needed to show she was poor in an area, to a therapist or school for example."

Other caregivers did not consider the PEDI-CAT (ASD) useful, as it provided "nothing new", produced results that were "misleading" or merely inaccurate: "Using just this tool alone, I do not feel [the assessment] accurately measured my [child's] actual challenges." As a result,

caregivers expressed concern regarding use of the measure in determining support needs: “If I'm having trouble [completing the PEDI-CAT (ASD)], when I see them 24/7, how can a professional who only sees them occasionally answer or be able to reflect upon results and make decisions?” Another caregiver stated, “It doesn't seem to have provided more evidence to support increased funding”.

Table 10

Summary of Caregiver-Reported Likes and Dislikes of the PEDI-CAT (ASD)

	Likes	Dislikes
Administration	Quick and easy.<br>Can be completed independently at own pace.<br>Computer-based and user-friendly.	Difficult to complete if child needs supervising.<br>Computer can be slow to load.
Structure/Format	Descriptions and examples provided.<br>Visual images.<br>Presentation of related questions in categories [domains] allowed focus on one area at a time making it less overwhelming.<br>Layout with one question per page.<br>Multiple-choice format.<br>Range of response options (including "I don't know" option).<br>Response options in 'Responsibility' section – capture relative amount of responsibility assumed by parent and child.	Layout of questions on the screen (little variation makes it difficult to sustain focus).<br>Font too small and difficult to read.<br>Response options – too few, not sensitive enough, unclear how to score noncompliance.<br>No opportunity to provide additional information and explanations.
Content	Comprehensively covers a broad range of day-to-day functioning.<br>Provides an expected level of functioning.<br>Includes content specific to autism.<br>Parent-friendly language and easy to understand questions.<br>Questions are non-invasive and task focused.	No consideration of context and functioning across different times and environments.<br>Not "inclusive" or "personalized" enough to capture differences.<br>American terminology (e.g., money references).<br>Some descriptions lacked explanation / clarification.<br>Questions are too general / vague.<br>Many questions are not age appropriate or otherwise irrelevant.<br>Too much repetition.

https://doi.org/10.1007/s10803-024-06366-7
